# Selections and Abstracts

**Published:** 1915-11

**Authors:** 


### SELECTIONS AND ABSTRACTS

### RESOLUTIONS ADOPTED BY THE AMERICAN MEDICAL EDITORS' ASSOCIATION.

Whereas, The American Medical Editors' Association believe that the principle of the freedom of the press bears unusual force in relation to the medical press, discussing subjects germane to medical progress, and

Whereas, the Southern California Practitioner has been indicted by the United States Postal Department because of the publication of an article dealing with the "sex question," which appeared in the issue of March, 1914.

Be it Resolved, that the American Medical Editors' Association express to Dr. George E. Malsbary, Editor of the Southern California Practitioner, its confidence and moral support in the pending action.

Be it Resolved, that the American Medical Editors' Association assures Dr. Malsbary of its willingness and readiness to afford him any assistance and support within its power according to the Constitution and By-Laws.

Ira S. Wile,

C. W. Fassett,

Henry R. Harrower,

Committee.

October 19, 1915.

---

### THE NECESSITY FOR CO-OPERATION BETWEEN THE VOCAL TEACHER AND LARYNGOLOGIST.*

Irving Wilson Voorhees, M. S., M. D., New York.

During the past year we have all felt within us a reflection of the indescribable suffering now going on across the great waste of waters. However we may try to put the thought of war out of our minds, it has risen before us like a specter in the night, it has struck depths of emotion and grief of which we were only dimly conscious heretofore, it has softened and humanized us.

And yet even war as horrible, as terrifying, as devastating as it is, has its amenities. There is in every great castastrophe, in every holocaust, however quickly and ruthlessly it swallows up the bodies and souls of men, a brighter side which in no sense completely balances or makes up for what is lost, but which serves to soothe the mind and heart and makes us better able to bear its heavy burden of woe. It is an illustration of how good may, after all, come out of evil.

But I would not sadden your hearts on this occasion by a recital of the woes of humanity. I intended merely to touch upon the war situation as it is likely to influence two great fields of human activity—music and medicine—medicine because that happens to be my chosen profession, and music because that is the profession to which most of you are devoted, and one in which I am, as you know, very greatly interested, particularly in its vocal aspect.

In both of these sciences, for music is a science as well as an art, we are obliged to hark back to old Europe for our historical and evolutionary bearings. For many years Paris was the one great medical centre. Here it was that Oliver Wendell Holmes and other famous physicians had their early training. It may be news to some of you to hear that Holmes was an ardent student and a great teacher of medical science, and yet we have numerous passages from his writings and glittering phrases from his note books showing how he strove and delved and worked both at the dissecting table and at the bedside during two years of residence in the famous French capital.

From Paris the medical center of Europe shifted far south to Vienna, that lazy, care-free metropolis, where I myself found inspiration for research in new fields and an overwhelming desire to cultivate many a neglected angle in fields that I thought were old. In Vienna, despite any apparent indifference to what we love to call the "mad rush" of New York or Chicago, there is a gospel of work that is infectious to the last degree. To that city have gone from cities and towns of our great West and South hundreds of men and women in pursuit of advanced work both in music and medicine. A casual observer will find them apparently doing nothing but enjoying "kaffee klatch" in mid-afternoon of a bright summer's day, but if you sit down and talk with them you will find that the hour spent there is for the most part an hour of reflection in which the past is carefully introspected and plans for the future mapped out with an all-absorbing enthusiasm.

So much for the superficial glimpse of the made-in-Europe variety of professional purpose. But we may reasonably ask, "How is this war going to affect the European cities as teaching centers?" Some say not at all, that as soon as the war is over there is going to be such in influx of American students as the Old World has never before seen. Of this I am doubtful. During these months of prohibitive globe-trotting I believe that America is going to be discovered anew, that the mantle of Elijah is going to fall upon Elisha anew, and that the nuclei of learning exemplified by so many of the good young minds in this country are going to divide and sub-divide until a new race, a new generation of enthusiastic students is grown on American soil and has taken firm and everlasting root here. For this we will need to change ou natural character somewhat. We need to be more serious in our efforts at home, to give up our careless sloven ways of speech, to turn a deaf ear to "ragtime" and other forms of prostituted art, to do our own thinking and to cultivate a greater sense of assurance in what we are capable of accomplishing. This seems to be a great and impossible task you say. I answer that there is no task too great or too impossible for young and vigorous America. Our energies are elemental, dynamic, intuitive. They seek expression in thousands of ways, blindly perhaps, yet ever ready to be brought out into the light and like light itself made radiant by a mysterious, inscrutable force. We have initiative in plenty, what we need is intelligent direction. I feel confident that with such potent forces in American music as Dvorak and Gustav Mahler, Karl Muck, Josef Stransky and others whose names are known to you all, we have an impetus toward the establishment of a selective and creative musical sense which in a few short years is destined to become traditional with us.

When I first began to pay attention to the vocal problem and came into closer contact with teachers of singing I was at a loss to understand the basis of antagonism which apparently exists in some quarters between the teacher and the laryngologist. Instead of a spirit of friendly co-operation there seemed to be a watchful and jealous eye under which the pupil was obliged to exist. Teachers were blaming nose and throat specialists for performing unnecessary operations, ruining voices, giving singing lessons, undermining the influence of the teacher over his pupil and of raising havoc generally. The laryngologist on his side indulged in disparaging remarks and showed other evidences of disregard for the members of what has come to be a profession closely akin to rhinoloryngology. In each and every case I found that the accusers had no intimate knowledge of each other's work, of the problems they were trying to solve or of the difficulties to be overcome. It was manifest that few physicians have ever visited a vocal studio to watch the actual work of instruction and that the teacher was equally uninformed of the specialists viewpoint in the management of nose and throat conditions.

One thing, however, is certain, namely, that both have at heart one common aim, one definite purpose—the welcome of the pupil. Many a singing teacher devotes hours and hours in an attempt to build up a voice which to the casual observes seems to offer little hope or promise, and this without one cent of reward. Unfortunately, too, such efforts are sometimes rewarded by no sense of gratitude on the pupil's part. On the contrary, one may see such utter lack of appreciation as to shake his faith in human nature. But the teacher, like the physician, takes it all as a part of the day's work and plods along doing his stint of work as best he can from day to day. When we think of the long apprenticeship, the hard struggle for recognition, the relatively short career and the rapid decline we must in all justice condone much in singers that would in others be quite inexcusable.

The burning question is after all, "How long is a voice going to last?" And again, "How can we help to lengthen this span of vocal life?" To begin with, we must consider each individual separately for his problem may be quite different from that of his associate or friend. Any voice which is going to amount to anything must be built up on a solid mental and physical basis. A beautiful tone quality is of little ultimate value if there are no brains to top it off with. Or given both of these in good measure one can expect little if the physical organism, the general health is vitally deficient. Mens sana in corpore sano is just as true of the singer as of any other person. In many respects it is of the utmost significance. To excel in song requires more natural talent, more careful adjustment of essentials than in any other art with which I am acquainted. Seriousness of purpose and hard work while important are far less so than talent or intelligence but we can correct many physical defects and can mould thought processes. First of all the body as a whole must be made an efficient instrument of the will. The physical organism must be put right. It is disappointing in the extreme to see the pupil struggle along for two or three years under the burden of nasal insufficiency, obstructive adenoids, or chronic disease of the tonsils and then become suddenly aware of the reason for lack of progress. All this for want of a thorough and careful physical examination at the outset. Every beginner should in my opinion undergo the same examination as if he were a candidate for a policy of life insurance. Every known method of determining his ability and capacity should be utilized. The general physical equipment should be investigated and where defective should be put in the best possible condition. The size, weight, strength, endurance, condition of muscles, nerves, digestion, etc., should be set down and any improvement noted at a later date for purpose of comparison.

The pupil is the clay with which we work. His impressions are easily moulded in the beginning and can be changed later on only with the greatest difficulty. His reliance upon teacher and physician is or ought to be absolute. Good advice will make him; bad advice will mar him and the career which is a part of him forever. He should not be told too much about anatomy and physiology, or of the movements of the jaw, tongue, lips, etc.—we must beware of the idee fixe teachers and voice specialists alike!

Every teacher and physician should know a great deal more than he is obliged to use in his daily work. The fountain of knowledge should be so inexhaustible that no pupil or patient can pump him dry. This applies of course to these things which are known of a certainty, not to the merely speculative and quixotic. There must be an increasing desire to explore the unknown, and to push back the veil of ignorance a little further, so that the physical horizon of the chosen field shall become a vanishing quantity. It is true that all roads lead to Rome, but there is certainly one which is wider, smoother, shorter and shadier than all the others. It is this which vocalists as a whole are seeking in the efforts now making for "standarization." Whether it can be done or not is an undecided question, but there is no harm in striving after it. Nothing but good can came out of discussions of the subject even if such discussions are sometimes attended with more heat than light. The light is needed to be sure, but the heat is a purifier and a refiner of ideas. There must be, after all, certain guiding principles, which are the basis of all successful vocal results, the differences are those of degree only and not of the fundamental fact.

Although the legitimate fields of laryngologist and vocal teacher are distinct and separate, they have so much in common that co-operation should prove of great advantage in the training of singers. Before a pupil begins active work he should be thoroughly tried out and examined by a laryngologist who knows something of the singing problem. The nose, throat and ears should be tested by every known method, and a definite idea should be obtained also of the general bodily health. It is helpful to know the range and compass of the voice, and to determine any serious faults of tone production which are not due to purely physical causes. For this one may try out a voice at the piano and make careful notes of what is found, then at a later date this can be done again for purpose of comparison. Any recommendation as to vocal exercises should be communicated to the teacher direct and not to the pupil, since it is the teacher who is immediately responsible for vocal growth and progress. The larnygologist should avoid falling into the error of always finding some physical cause for every disturbance of which the singer complains. Very often such symptoms are the result of something the singer does in practice at home which is not found out at once by the teacher. There are, too, some cases which do not develop satisfactorily where neither the teacher nor the physician can find any discoverable cause for the defect. Secretory changes in the mucous membrane are of especial importance and are frequently overlooked. There may be an abundant sticky secretion which dries and forms crusts in the nose, or the mucous membrane may be very dry from glandular inactivity—a condition which interferes greatly with the singers finer work, especially the soft tones.

It is not my purpose to go into these matters exhaustively, but merely to point out a common ground upon which the rhilologist and vocal teacher can think, act and work together for the furtherance of perfection in the science and art of song. Selected.

